# Altered circulating GDF-15 level predicts sex hormone imbalance in males with major depressive disorder

**DOI:** 10.1186/s12888-023-04527-z

**Published:** 2023-01-12

**Authors:** Ying Li, Ting Mei, Ting Sun, Xuan Xiao, Rui Peng

**Affiliations:** 1 Department of Clinical Laboratory, Renmin Hospital of Wuhan University, 238 Jiefang Road, Wuhan, China; 2 Eye Central, Renmin Hospital of Wuhan University, Wuhan, China; 3 Nursing Department, Wuhan No.1 Hospital, Wuhan, China

**Keywords:** Sex hormones, Imbalance, Growth differentiation factor 15, Major depressive disorder

## Abstract

**Background:**

It has been hypothesized that higher growth differentiation factor 15 (GDF15) level and lower testosterone/ estradiol (T/E) ratio are associated with major depressive disorder (MDD), yet the underlying effect of serum GDF15 on hinting the T/E ratio imbalance is not fully understood. We observed the correlation between serum T/E ratio and circulating GDF15 in male depressed cohort.

**Methods:**

The sample consisted of participants (aged 18 ~ 65 years) from the Renmin Hospital of Wuhan University with MDD (*n* = 412) defined according to a Structured Clinical Interview for DSM-V (SCID), and male healthy controls (*n* = 137). Serum levels of testosterone, estradiol, and depression risk biomarkers (thyroid hormone, lipids, hs-CRP, Tenascin-C [TNC], GDF15, KLF4, Gas6, and sgp130) were measured. The associations among log-transformed T/E ratio and these biomarkers were analyzed using univariate correlation analysis, category analyses, and linear regression adjusting for standard risk factors.

**Results:**

Of the sample, 36.89% had lower T/E ratio (< 10:1) and 10.20% had higher T/E ratio (> 20:1). After multivariable adjustment, T/E ratio was negatively associated with GDF15 (-0.095 [95% *CI* -0.170 ~ -0.023] standard deviation [SD] change per SD increase in lg[T/E], *P* = 0.015) and inversely related to TNC (-0.085 [95% *CI* -0.167 ~ 0.003] standard deviation [SD] change per SD increase in lg[T/E], *P* = 0.048). Serum T/E ratio was negatively associated with GDF15 level in both FT3, TSH and HDL strata, whereas this association was not observed in TNC. In T/E ratio strata analyses, there is a significant and negative correlation among T/E ratio and GDF15 in depressive patients with sex hormone imbalance, yet this relationship was not investigated in patients with sex hormone balance.

**Conclusion:**

In our community-based observation, circulating GDF-15 level was greatly and inversely associated with serum T/E ratio, indicating that higher GDF-15 alerts sex hormone imbalance in patients with MDD.

**Supplementary Information:**

The online version contains supplementary material available at 10.1186/s12888-023-04527-z.

## Background

Major depressive disorder (MDD) is considered as a major public healthy challenge and is predicted to the significant cause of disability worldwide in 2020 [1]. This disorder leads to the soaring costs for treatment and heavy mental pressure for patients [2]. Depressive etiology and pathophysiology have been poorly demonstrated. Research advances in recent decade demonstrated the potential effect of new biomarkers which may impact mood in combination with the more neuroendocrine mechanisms. A large number of observations have proposed that dysregulation of neurotransmitter, dysfunction of hypothalamic-pituitary-thyroid (HPT) and inflammatory response are closely associated with the development of MDD [3–5]. Furthermore, extensive investigations have demonstrated that sex hormone deficiency may be involved in the pathogenesis of depression [6–8]. Thus, sex hormone deficiency might be involved only in specific subgroups of depression due to the complex interactions of social, psychological and biological factors in depression. A basic study has shown that both testosterone and estradiol have anxiolytic- and antidepressant-like effects in gonadectomized male animals, and the protective effects of testosterone are mediated by its aromatization [9]. The excess aromatase activity could lead to low testosterone and relatively increased estradiol levels (sex hormone imbalance) in men [10], and sex hormone imbalance was found to be correlated with the occurrence of depressive disorder [11].

Recent studies have displayed that many serum biomarkers, such as Tenascin-c (TNC), GDF-15, growth arrest-specific 6 (Gas6), kruppel like factor 4 (KLF4), and soluble glycoprotein 130 (sgp130), were found to be potential associated with MDD. Some researchers demonstrated that serum elevated TNC and GDF15 levels may be inflammatory biomarkers for depressive pathophysiology [12, 13]. Gas6 deficiency may be a potential mediator for MDD via increasing oligodendrocyte loss and microglial activation [14]. Whereas KLF4 could induce microglial activation through increasing the production of inflammatory cytokines [15]. An earlier study also reported that sgp130 may be a therapeutic target in chronic depression [16]. Moreover, related studies have demonstrated that gonadal hormone are closely associated with these serum biomarkers. Our research team have confirmed that testosterone is significantly and positively associated with serum Gas6 level [17], and serum sgp130 was positively correlated with estradiol and testosterone/estradiol ratio in male patients with coronary atherosclerotic disease [18]. TNC, KLF4 and GDF15 were also found to be associated with sex hormone levels in earlier studies [19, 20]. However, the associations between sex hormone and these biomarkers in depressive patients remain poorly demonstrated, and the alteration in which marker could alert sex hormone imbalance in MDD is unclear.

In the current study, we explored the associations among serum T/E ratio and circulating depression risk biomarkers in depressed cohort. The main aim of this study was to select a biomarker to alert the sex hormone imbalance in depressive patients.

## Material and methods

### Study population

All male participants (412 depressive patients and 137 healthy controls) were enrolled form Department of Psychiatry at Renmin Hospital of Wuhan University. All subjects received a Structured Clinical Interview for DSM-V (SCID). In addition, Hamilton Depression Rating Scale (HDRS) and the Beck's Suicidal Ideation Scale (SSI) were also performed to assess the severity of depressive symptoms. Depressed symptoms were evaluated basing on SCID score at the first day of admission, and the background materials were recorded simultaneously. Moreover, the healthy controls enrolled in our study also receive the“physical exam and psychiatric interview” and their score within normal limits. Sociodemographic characteristics contained age and years of education. The following lifestyle and health indicators were evaluated: smoking status, alcohol use, and body mass index. Current alcohol use was evaluated by the Timeline Followback to assess alcohol use quantity and frequency over the past 30 days [21]. Clinical characteristics (duration of symptoms and age of onset) were also recorded. To determine a potential pathophysiological effect of treatment, antidepressants use was evaluated according to consulting the patients and family members, and medicine were included selective serotonin reuptake inhibitors (SSRI), serotonin-norepinephrine reuptake inhibitors (SNRI) and tricyclic antidepressants (TCA). The Medical Ethics Review Committee of Renmin Hospital of Wuhan University approved this study protocol, and all subjects provided written informed consent.

### Sample collection

Blood specimens were drawn from antecubital vein of all participants after an overnight fast. Serum samples were immediately centrifuged, recovered into 2 ml cryogenic vials and frozen at -80 ℃ until assays for various analyses.

### Depression risk biomarkers

Circulating concentrations of TNC, GDF15, KLF4, Gas6 and sgp130 were detected along with a panel of other biomarkers, including testosterone, estradiol, thyroid hormone, lipids, and hs-CRP, as detailed previously [22]. In brief, the concentrations of total cholesterol (TC), triglyceride (TG), high density lipoprotein cholesterol (HDL-C), low density lipoprotein cholesterol (LDL-C), and high-sensitivity C-reactive protein (hs-CRP) were measured using a Siemens Advia 2400 automatic biochemistry analyzer (Siemens, Erlangen, Germany). Free thyroxine (FT4), free triiodothyronine (FT3), thyroid-stimulating hormone (TSH), testosterone (T), and estradiol (E) concentrations were determined using a Siemens Advia Centaur CP (Siemens, Erlangen, Germany). The T/E ratio was calculated according to the values of testosterone and estradiol. TNC concentration was detected using an enzyme-linked immunosorbent assay with a detection limit of 0.78 ng/ml (Wuhan, China, CUSABIO), GDF15 level was measured by an enzyme-linked immunosorbent assay with a detection limit of 7.8 pg/ml (Wuhan, China, CUSABIO), KLF4 concentration was detected using an enzyme-linked immunosorbent assay with a detection limit of 18.75 pg/ml (Wuhan, China, CUSABIO), Gas6 content was measured by an enzyme-linked immunosorbent assay with a detection limit of 0.78 ng/ml (Wuhan, China, CUSABIO), sgp130 concentration was determined by an enzyme-linked immunosorbent assay with a detection limit of 0.25 ng/ml (Wuhan, China, Elabscience). All biomarker measurements were conducted in accordance with kit manufacturer standards.

### Statistical analyses

Variables were described as mean ± s.d. or percentages, and analyzed using SPSS software 20.0. All biomarkers were natural logarithmically transformed (to normalize their skewed distribution) and standardized. Differences in clinical characteristics were analyzed using χ2-tests or analyses of variance as appropriate. Difference in depression risk biomarkers were tested according to classification of T/E ratio using ANOVA. The associations among T/E ratio and depression risk biomarkers were assessed using single-factor correlation analysis and multivariable linear regression analysis. Stratified analysis was performed to further analyze the correlations between T/E ratio and GDF15, TNC in age, duration, FT3, TSH, HDL and hs-CRP strata.

## Results

### Study population

A total of 547 subjects, with a average age of 35.89 years (± 13.57 of s.d.), had complete epidemiologic inquiry and biomarkers measurements from the case–control study. The mean of duration of disease in depressive patients was 36.47 months (± 31.72 of s.d.), the average age of onset was 29.34 years (± 13.52 of s.d.), and 41.01% of the depressed patients comorbided anxiety. The analyses of depressive severity showed that more than half the patients suffered from moderate depression (Table 1).

**Table 1:** Characteristics of the study population

Characteristics	Controls (*n* = 137)	Depressive patients (*n* = 412)	*P*
Sociodemographic			
Age (years) (mean ± s.d.)	33.29 ± 10.71	36.48 ± 15.73	0.584
Education (years) (mean ± s.d.)	14.05 ± 3.41	12.92 ± 3.26	0.492
Lifestyle and health indicators			
Smoking status (%)			0.546
Non smoker	70.07	67.96	
Current smoker	29.93	32.04	
Alcohol use (%)	12.41	18.70	0.054
Body mass index (%)			**0.037**
Normal	67.15	59.47	
Overweight	23.36	30.10	
Obesity	9.49	10.43	
Clinical characteristics			
Duration (month) (mean ± s.d.)	-	36.47 ± 31.72	
Age of onset (years) (mean ± s.d.)	-	29.34 ± 13.52	
Comorbid anxiety (%)	-	41.01	
Antidepressant use (%)			
No antidepressant	-	72.33	
SSRI	-	10.68	
SNRI	-	12.62	
TCA	-	4.37	
Severity of depression			
Mild	-	29.61	
Moderate	-	51.21	
Severe	-	19.18	
Testosterone (T, ng/ml)	439.53 ± 136.08	331.64 ± 149.37	**< 0.001**
Estradiol (E, pg/ml)	31.08 ± 10.34	29.34 ± 11.82	0.367
T/E ratio	16.32 ± 5.94	12.17 ± 5.62	**< 0.001**
FT3 (pg/ml)	3.48 ± 0.31	3.24 ± 0.39	**0.006**
FT4 (ng/ml)	1.24 ± 0.13	1.26 ± 0.23	0.426
TSH (μIU/ml)	2.28 ± 0.88	1.71 ± 0.96	**< 0.001**
hs-CRP (mg/l)	0.38 ± 0.36	1.16 ± 3.06	**0.009**
TC (mmol/l)	4.11 ± 0.79	4.23 ± 0.83	0.392
TG (mmol/l)	1.16 ± 0.49	1.51 ± 0.57	**< 0.001**
HDL (mmol/l)	1.28 ± 0.26	1.16 ± 0.24	**0.024**
LDL (mmol/l)	2.24 ± 0.62	2.31 ± 0.71	0.604
TNC (ng/ml)	6.92 ± 2.87	11.79 ± 6.06	**< 0.001**
GDF15 (pg/ml)	52.22 ± 42.86	74.60 ± 64.37	**< 0.001**
KLF4 (pg/ml)	23.51 ± 13.86	13.12 ± 6.84	**< 0.001**
Gas6 (ng/ml)	6.07 ± 3.12	4.88 ± 2.43	**0.019**
sgp130 (ng/ml)	14.04 ± 4.13	13.54 ± 4.39	0.297

The biochemical variables of the participants are displayed in Table 1. The values of serum T, T/E ratio, FT3, TSH, HDL, KLF4 and Gas6 level in depressed patients were significantly decreased compared with healthy controls ( all *P* < 0.05). Whereas the serum levels of hs-CRP, TG, TNC and GDF15 in depressed subjects were higher than healthy controls ( all *P* < 0.05). The serum levels of E, FT4, TC, LDL and sgp130 unchanged in patients when compared to controls (all *P* > 0.05). Moreover, The associations among these biomarkers and severity of depression were shown in Supplemental Table 1. The results demonstrated that T/E ratio and GDF15 were closely associated with severity of depression.

### Altered biomarkers concentrations in different classification of serum T/E ratio

Based on extensive investigations, many researchers proposed a cut-point of 10 as the lower limit of normal T/E ratio, and a cut-point of 20 as the higher limit of normal T/E ratio in male [23]. In this study, one third of the depressed patient (36.89%) had lower T/E ratio (< 10:1) and 10.20% had higher T/E ratio (> 20:1). The proportion of participants with with lower T/E ratio increased from healthy controls (13.14%) to those with depression (36.89%). Table 2 shows the changes of depression risk biomarkers among lower, normal, and higher T/E ratio assessed by one-way analysis of variance. As shown in Table 2, the significant changes of FT3, GDF15 and Gas6 levels between lower, normal, and higher T/E ratio were observed (*P* < 0.05), whereas the levels of other risk factors unchanged significantly (*P* > 0.05). Higher levels of FT3 and Gas6 were investigated for increasing T/E ratio, and lower level of GDF15 was observed for increasing T/E ratio.

**Table 2:** Unadjusted means of different biomarkers according to T/E status

Variable	lgT/E status			*F*	*P*
	T/E < 10:1 (*n* = 152)	10:1 ≤ T/E ≤ 20:1 (*n* = 218)	20:1 < T/E (*n* = 42)		
Age	36.87 ± 15.90	35.19 ± 14.02	32.57 ± 12.26	0.996	0.370
FT3	3.22 ± 0.45	3.29 ± 0.42	3.48 ± 0.42	5.616	**0.004**
FT4	1.22 ± 0.18	1.23 ± 0.21	1.27 ± 0.18	1.139	0.321
TSH	1.90 ± 1.16	1.95 ± 1.34	1.98 ± 0.86	0.541	0.582
hs-CRP	0.80 ± 1.85	0.94 ± 2.61	0.60 ± 2.64	2.697	0.069
TC	4.07 ± 0.84	4.21 ± 0.96	4.31 ± 0.98	0.928	0.396
TG	1.31 ± 0.86	1.44 ± 1.28	1.80 ± 1.42	1.685	0.187
HDL	1.18 ± 0.30	1.15 ± 0.30	1.19 ± 0.30	0.415	0.660
LDL	2.22 ± 0.68	2.31 ± 0.77	2.20 ± 0.73	0.270	0.763
TNC	9.40 ± 5.18	8.75 ± 5.15	8.18 ± 4.49	1.752	0.175
GDF15	107.52 ± 62.15	83.55 ± 61.44	69.36 ± 61.67	8.827	**< 0.001**
KLF4	213.17 ± 258.73	210.41 ± 252.37	212.06 ± 311.12	0.197	0.821
Gas6	3.64 ± 2.51	4.34 ± 2.58	4.88 ± 3.30	3.458	**0.033**
sgp130	12.83 ± 4.38	14.06 ± 4.26	14.31 ± 4.04	0.617	0.542

### Correlations among log-transformed T/E ratio and biomarkers in MDD

Univariate correlation analysis was performed to study the associations between log-transformed T/E ratio and depression risk biomarkers in depressive participants. The results in Table 3 demonstrate that T/E ratio is positively and significantly associated with FT3 (*β* = 0.381, *P* < 0.05) and Gas6 (*β* = 0.061, *P* < 0.05); whereas T/E ratio is negatively and significantly associated with TNC (*β* = -0.075, *P* < 0.05) and GDF15 (*β* = -0.113, *P* < 0.001).

**Table 3:** Correlations of log-transformed T/E ratio and biomarkers

Variable	T/E		
	*β*	*T*	*P*
Age	0.023	0.378	0.706
FT3	0.381	2.173	**0.030**
FT4	0.048	0.311	0.756
TSH	0.013	0.348	0.728
hs-CRP	0.001	0.096	0.923
TC	0.045	0.020	0.731
TG	0.076	1.547	0.123
HDL	-0.150	-1.363	0.174
LDL	-0.056	-0.661	0.509
TNC	-0.075	-2.011	**0.045**
GDF15	-0.113	-4.161	**< 0.001**
KLF4	-0.012	-0.713	0.477
Gas6	0.061	1.980	**0.048**
sgp130	0.029	0.241	0.810

### Logistic regression analyses for log-transformed T/E ratio and GDF15

Results from multivariate linear regression models relating log-transformed T/E ratio to depression risk biomarkers are displayed in Table 4. According to the results of univariate correlation analysis, we incorporated the age, FT3, HDL, TG, Gas6, GDF15 and TNC to perform the multivariable logistic regression analyses. Upon multivariable adjustment for age, FT3, HDL, TG and Gas6, higher T/E ratio remained statistically significantly related to lower GDF15 level (estimate -0.095 [95% *CI* -0.170 ~ -0.023] standard deviation [SD] change per SD increase in lg[T/E], *P* = 0.015) and TNC (estimate -0.085 [95% *CI* -0.167 ~ 0.003] standard deviation [SD] change per SD increase in lg[T/E], *P* = 0.048). In addition, upon multivariable adjustment for only age, higher T/E ratio remained statistically significantly related to lower GDF15 level (estimate -0.118 [95% *CI* -0.168 ~ -0.075] standard deviation [SD] change per SD increase in lg[T/E], *P* = 0.001) and TNC (estimate -0.087 [95% *CI* -0.157 ~ -0.019] standard deviation [SD] change per SD increase in lg[T/E], *P* = 0.009).

**Table 4:** Adjusted associations between log-transformed T/E ratio, GDF15 and TNC

	GDF15			TNC		
	*β*	*95% CI*	*P*	*β*	*95% CI*	*P*
Model1	-0.095	-0.170 ~ -0.023	**0.015**	-0.085	-0.167 ~ 0.003	**0.048**
Model2	-0.095	-0.169 ~ -0.025	**0.009**	-0.081	-0.158 ~ -0.001	**0.041**
Model3	-0.118	-0.168 ~ -0.075	**0.001**	-0.087	-0.157 ~ -0.019	**0.009**

Model 1: adjusted for age, FT3, HDL, TG and Gas6; Model 2: adjusted for age, FT3 and HDL; Model 3: adjusted for age

### Associations between log-transformed T/E ratio and GDF15 across risk categories

Table 5 presents correlation coefficient for the association among log-transformed T/E ratio and GDF15, TNC levels, stratified by age, duration, FT3, TSH, HDL and hs-CRP. Inverse associations among T/E ratio and GDF15 level were found for all FT3, TSH and HDL strata, whereas the negative relations between T/E ratio and GDF15 level were noted for partial age, duration and hs-CRP strata. However, the inverse relations among T/E ratio and TNC level in all age, duration, FT3, TSH, HDL and hs-CRP strata were not observed.

**Table 5:** Correlations of log-transformed T/E ratio, GDF15 and TNC across categories of age, duration, FT3 level, TSH level, HDL level and hsCRP level

	GDF15			TNC		
	*β*	*T*	*P*	*β*	*T*	*P*
Age						
≥ 45	-0.090	-1.739	0.085	-0.059	-0.945	0.347
45 ~ 25	-0.144	-3.925	**< 0.001**	-0.025	-0.449	0.654
≤ 25	-0.081	-1.391	0.167	-0.143	-1.882	0.062
Duration						
≥ 3	-0.043	-0.855	0.394	-0.089	-1.426	0.156
< 3	-0.124	-2.511	**0.013**	-0.040	-0.769	0.443
FT3						
≥ 3.32	-0.084	-2.232	**0.027**	-0.086	-1.530	0.128
< 3.32	-0.126	-2.961	**0.003**	-0.089	-1.794	0.074
TSH						
≥ 1.717	-0.135	-3.105	**0.002**	-0.064	-1.254	0.211
< 1.717	-0.092	-2.639	**0.009**	-0.084	-1.503	0.134
HDL						
≥ 1.01	-0.126	-2.795	**0.006**	-0.062	-1.253	0.212
< 1.01	-0.067	-2.053	**0.042**	-0.129	-2.304	**0.022**
hs-CRP						
≥ 0.11	-0.037	-0.084	0.286	-0.051	-1.224	0.222
< 0.11	-0.180	-4.480	** < 0.001**	-0.098	-1.506	0.133

### Altered GDF-15 level alerts sex hormone imbalance in MDD

In order to further demonstrate whether the altered serum GDF15 level could alert sex hormone imbalance in depressive subjects, we analyzed the associations between log-transformed T/E ratio and depression risk biomarkers across the strata of T/E ratio. Based on the earlier report [23], the normal T/E ratio in men was defined as 10:1 ~ 20:1 (sex hormone balance), and the T/E ratio lower than 10:1 or more than 20:1 were considered as abnormal (sex hormone imbalance). As shown in Table 6, there is a significantly and negatively correlation between T/E ratio and GDF15 level in depressed patients with sex hormone imbalance. These findings further verify that increased circulating GDF15 level could alert sex hormone imbalance in MDD.

**Table 6:** Correlations of log-transformed T/E ratio and biomarkers across categories of sex hormone balance and unbalance

	Balance			Unbalance		
	*β*	*T*	*P*	*β*	*T*	*P*
FT3	-0.090	-0.815	0.416	0.808	2.615	**0.010**
FT4	-0.238	-2.740	**0.007**	0.495	1.619	0.107
TSH	-0.001	-0.047	0.963	0.052	0.674	0.501
hs-CRP	0.005	0.607	0.544	-0.021	-0.813	0.418
TC	-0.124	-1.564	0.120	0.116	0.522	0.602
TG	-0.032	-1.039	0.301	0.166	2.136	**0.034**
HDL	-0.042	-0.606	0.546	-0.163	-0.911	0.364
LDL	-0.092	-1.832	0.069	-0.083	-0.575	0.566
TNC	-0.012	-0.549	0.584	-0.126	-1.656	0.099
GDF15	0.006	0.355	0.723	-0.193	-4.001	**< 0.001**
KLF4	-0.018	-1.686	0.094	-0.006	-0.207	0.836
Gas6	0.006	0.316	0.752	0.056	1.102	0.272
sgp130	-0.094	-1.192	0.239	0.168	0.618	0.541

## Discussion

In a host of depressive patients and healthy controls, to our knowledge the first of its kind, we demonstrated that after adjusting a large number of potential confounding factors, high serum abundance of GDF15 was associated with sex hormone imbalance in MDD. Not only the overall correlation between T/E ratio and GDF15 level was of significant effect size, but also the effect size was more pronounced in depressed patients with the sex hormone imbalance. Clinically higher GDF15 level was more likely to be observed in depressive individuals with lower T/E ratio. In addition, the association among T/E ratio and TNC level was of rather small effect size relative to GDF15. In the current study, we also observed that being depression was correlated to lower FT3 and TSH levels, in line with previous studies [22, 24]. Hyperparathyroidism has been regarded as the most important mechanism whereby inflammatory response could lead to the development of MDD.

At the molecular level, GDF15 was found to be a stress-responsive cytokine which is secreted by multiple cells, such as macrophages, endothelial cells, and neurons in response to oxidative stress, injury, and inflammation [25, 26]. GDF15 acts as a growth factor as well as immunomodulator, and has been thought to be involved in cognitive decline [27, 28]. Our study demonstrated that GDF15 level in depressed subjects was significantly higher than healthy controls, as well as increased GDF15 is closely associated with sex hormone imbalance in patients with MDD. Whereas the association between GDF15 and T/E was not observed in healthy controls. In prior studies, GDF15 was significantly and inversely associated with testosterone level in subjects with anaemia [21], as well as estradiol could activate GDF15 expression in the tamoxifen resistant cell systems [29]. These previous investigations confirm our results that T/E ratio was closely significantly and negatively associated with circulating GDF15 level in depressed patients. There was a demonstration that GDF15 may participant in the etiology of Parkinson's disease through the activation of chemokine receptor 4 (CXCR4) [30]. GDF15 has also been reported to promote simultaneous astrocyte remodeling and tight junction strengthening at the blood–brain barrier (BBB), which is closely associated with the occurrence and development of depression [31]. Moreover, it was reported that chronic depletion of gonadal testosterone results in BBB dysfunction and inflammation in male mice to induce depressive behavior [32]. According to above findings, it supported that GDF15 and sex hormone involve in the development of MDD. In addition, our results also showed that both GDF15 upregulation and T/E ratio decreasing were positively associated with the severity of MDD.

The present research possesses several limitations. First, as the research design was mainly cross-sectional, on deduction on the directionality of the correlation could be performed. We could not adjust the determined correlation for dietary intake of cholesterol, which can be metabolized into testosterone and estradiol, because nutritional information can not be collected. Whereas only small part of serum testosterone is detected by dietary intake, and high intake of saturated fat could result in the elevation of estradiol [33]. Furthermore, soy intake was found to impact the secretion or metabolism of sex steroids and further alter the circulating levels of testosterone and estradiol [34]. Then the the medicine used for 1–2 months prior to recruitment may affect the sex hormone and depression risk biomarkers [35]. Therefore, we were also not able to adjust the relationship for drug treatment of antidepressants. In addition, this research has several crucial superiorities, such as adjustment for possible confounders, multilevel analyses for observed association.

Collectively, our results afford compelling evidence to previous studies, which mainly investigated the alterations in sex hormone or single risk biomarkers in MDD. Remarkably, after adjustment for multiple risk factors, serum T/E ratio was closely and inversely associated with GDF15, suggesting that the sex hormone imbalance in vivo may contribute to the increased circulating GDF15 level. Adjusting for those confounders did not attenuate the correlation among serum T/E ratio and GDF in MDD. This finding should induce further longitudinal and basic experimental test stating consistent time-sequenced correlations to investigate causality in the sex hormone-GDF15 link in MDD. Confirming an involvement of GDF15 in the pathway to depressive development due to sex hormone imbalance may have important implications. GDF15 could possesses positive and negative roles depending on the status of cells and their environment [36]. We speculate that individuals with sex hormone imbalance could stimulate GDF15 expression, and it shows a negative effect to promote the development of depression, including the induction of inflammation and oxidative stress [37, 38]. A previous study has demonstrated that GDF15 could regulate the expression of relevant genes for the hormone dependent regulation in advanced metastatic castration-resistant prostate cancer, including androgen receptor (AR) and aromatase gene [39]. Therefore, it supported that GDF15 upregulation induces sex hormone imbalance in males with MDD. Addressing these problem successfully could help us to thought whether normalization of serum T/E ratios may become a possible future strategy to prevent or treat MDD. Furthermore, normalization of serum GDF15 level may be considered as a potential biomarker to hint sex hormone imbalance in vivo.

In summary, circulating GDF15 level was greatly and inversely associated with serum T/E ratio, indicating that higher GDF15 alerts sex hormone imbalance in patients with MDD. Moreover, our results may indicate that GDF15 induces the expression of aromatase to metabolize testosterone transformation to estradiol and leads to sex hormone imbalance, which further induces the development of MDD through some signaling pathways.

## Supplementary Information

**Additional file 1:** **Supplemental Table 1.** Associations among biomarkers and severity of depression.

## Data Availability

The datasets used and/or analyzed during the current study available from the corresponding author on reasonable request.

## Abbreviations

MDD: Major depressive disorder
TNC: Tenascin-c
GDF15: Growth differentiation factor 15
Gas6: Growth arrest-specific 6
KLF4: Kruppel like factor 4
sgp130: Soluble glycoprotein 130
E: Estradiol
T: Testosterone
TSH: Thyroid-stimulating hormone
FT3: Free triiodothyronine
FT4: Free thyroxine
hs-CRP: High-sensitivity C-reactive protein
LDL-C: Low density lipoprotein cholesterol
HDL-C: How density lipoprotein cholesterol
TG: Triglyceride
TC: Total cholesterol
TCA: Tricyclic antidepressants
SNRI: Serotonin-norepinephrine reuptake inhibitors
SSRI: Selective serotonin reuptake inhibitors
SCID: Structured clinical interview for DSM-IV
HDRS: Hamilton depression rating scale
SSI: Beck's suicidal ideation scale
