# Supplementary material for: Altered circulating GDF-15 level predicts sex hormone imbalance in males with major depressive disorder
Source: BMC Psychiatry. 2023 Jan 12;23:28. doi: 10.1186/s12888-023-04527-z (PMC9835386; doi:10.1186/s12888-023-04527-z)
Supplement: Supplementary file 1 — Additional file 1: Supplemental Table 1. Associations among biomarkers and severity of depression. [file 12888_2023_4527_MOESM1_ESM.doc]

**Supplemental Table 1**. Associations among biomarkers and severity of depression

| Characteristics | Severity of depression | | | *F* | *P* |
| --- | --- | --- | --- | --- | --- |
|  | Mild (n=122) | Moderate (n=211) | Severe (n=79) |  |  |
| Sociodemographic |  |  |  |  |  |
| Age (years) (mean±s.d.) | 36.12±15.18 | 35.54±14.38 | 34.68±14.44 | 0.236 | 0.790 |
| Education (years) (mean±s.d.) | 13.25±3.50 | 14.85±4.03 | 13.95±3.49 | 0.312 | 0.527 |
| Lifestyle and health indicators |  |  |  |  |  |
| Smoking status (%) |  |  |  | 0.255 | 0.725 |
| Non smoker | 65.57 | 71.09 | 63.29 |  |  |
| Current smoker | 34.43 | 28.91 | 36.71 |  |  |
| Alcohol use (%) | 22.95 | 15.62 | 20.25 | 0.185 | 0.827 |
| Body mass index (%) |  |  |  | 2.495 | **0.042** |
| Normal | 55.74 | 62.09 | 58.23 |  |  |
| Overweight | 33.61 | 31.28 | 21.52 |  |  |
| Obesity | 10.65 | 6.63 | 20.25 |  |  |
| Clinical characteristics |  |  |  |  |  |
| Duration (month) (mean±s.d.) | 34.26±28.54 | 36.97±32.85 | 36.15±31.52 | 0.296 | 0.912 |
| Age of onset (years) (mean±s.d.) | 30.21±12.58 | 28.99±13.47 | 29.84±13.68 | 0.412 | 0.256 |
| Comorbid anxiety (%) | 42.62 | 46.45 | 24.05 | 0.987 | 0.064 |
| Antidepressant use (%) |  |  |  | 3.654 | **0.011** |
| No antidepressant | 80.33 | 78.20 | 44.30 |  |  |
| SSRI | 5.74 | 7.58 | 26.58 |  |  |
| SNRI | 8.20 | 11.37 | 21.52 |  |  |
| TCA | 5.73 | 2.85 | 7.60 |  |  |
| T[estosterone](../../../../C:/Users/lenovo/AppData/Local/youdao/dict/Application/8.3.1.0/resultui/html/index.html#/javascript:;) (T, ng/ml) | 360.98±16.09 | 345.33±152.09 | 335.84±139.77 | 0.727 | 0.484 |
| E[stradiol](../../../../C:/Users/lenovo/AppData/Local/youdao/dict/Application/8.3.1.0/resultui/html/index.html#/javascript:;) (E, pg/ml) | 28.17±13.00 | 30.89±13.38 | 31.07±12.84 | 1.904 | 0.150 |
| T/E ratio | 14.51±8.28 | 12.48±5.20 | 10.84±0.63 | 10.008 | **0.000** |
| FT3 (pg/ml) | 3.31±0.42 | 3.27±0.46 | 3.29±0.40 | 0.321 | 0.726 |
| FT4 (ng/ml) | 1.23±0.16 | 1.21±0.21 | 1.26±0.20 | 1.952 | 0.143 |
| TSH (μIU/ml) | 2.01±1.11 | 1.91±1.36 | 1.88±1.03 | 0.302 | 0.740 |
| hs-CRP (mg/l) | 0.68±1.92 | 0.99±2.77 | 0.76±1.71 | 0.717 | 0.489 |
| TC (mmol/l) | 4.05±0.76 | 4.16±0.99 | 4.27±0.87 | 1.069 | 0.345 |
| TG (mmol/l) | 1.43±0.93 | 1.38±1.26 | 1.45±1.02 | 0.109 | 0.897 |
| HDL (mmol/l) | 1.14±0.28 | 1.18±0.31 | 1.16±0.29 | 0.450 | 0.638 |
| LDL (mmol/l) | 2.19±0.63 | 2.26±0.78 | 2.36±0.72 | 0.966 | 0.382 |
| TNC (ng/ml) | 8.90±5.07 | 8.85±5.06 | 9.17±5.29 | 0.112 | 0.894 |
| GDF15 (pg/ml) | 78.98±59.29 | 90.72±65.53 | 112.42±60.57 | 6.038 | **0.000** |
| KLF4 (pg/ml) | 217.13±260.91 | 196.79±250.72 | 243.38±280.54 | 0.770 | 0.464 |
| Gas6 (ng/ml) | 4.14±3.01 | 4.03±2.50 | 4.29±2.50 | 0.248 | 0.780 |
| sgp130 (ng/ml) | 12.83±4.42 | 13.79±4.30 | 15.13±3.71 | 1.379 | 0.257 |
